# Risk of CNS relapse following pathological complete response to neoadjuvant chemotherapy in early breast cancer

**DOI:** 10.1007/s10549-026-07915-7

**Published:** 2026-03-23

**Authors:** Luciana de Moura Leite, Guilherme Rossato de Almeida, Monique Celeste Tavares, Marcelle Goldner Cesca, Fernando Augusto Batista Campos, Fernanda Alves de Oliveira, Debora Maciel Santana Dornellas, Erick F. Saldanha, Paula Tavares Guimarães, Daniella Dias Sá de Arruda, Maria Fernanda Simões Devides de Held, Rafael Lima Viana, Francisca Giselle Rocha Moura, Simone Klug Loose, Sinara Figueiredo Silva, Rafaela Pirolli, Camilla Albina Zanco Fogassa, Bruna Raphaeli Silva Mattos, Solange Moraes Sanches, Vladmir C. Cordeiro de Lima

**Affiliations:** 1 Department of Medical Oncology, A.C. Camargo Cancer Center, São Paulo, SP Brazil; 2 Department of Medical Oncology, Instituto Do Câncer de São Paulo (ICESP), São Paulo, SP Brazil; 3 Department of Medical Oncology, Hospital Israelita Albert Einstein, São Paulo, SP Brazil; 4 Division of Medical Oncology and Hematology, Princess Margaret Cancer Center, University Health Network, University of Toronto, Toronto, Canada

**Keywords:** Neoadjuvant chemotherapy, Pathologic complete response, Central nervous system metastases

## Abstract

**Purpose:**

Pathological complete response (pCR) after neoadjuvant chemotherapy (NAC) improves outcomes in breast cancer (BC); however it may not prevent brain metastases. We evaluated central nervous system (CNS) recurrence patterns in early-stage BC following NAC according to pCR.

**Methods:**

All consecutive stage I–III BC treated with NAC and surgery at a single center between 2007 and 2018 were analyzed. Endpoints included the impact of pCR on CNS recurrence across BC subtypes—hormone receptor-positive(HR)/HER2-negative, HER2-positive and triple-negative (TNBC), CNS recurrence patterns and overall survival (OS) after CNS relapse. Statistical comparisons included Fisher’s Exact test, Chi-square, Kaplan–Meier, and regression analyses.

**Results:**

Among 1147 patients, 537 had HR-positive/HER2-negative, 301 HER2-positive, 309 TNBC, mostly stage III, treated with anthracycline + taxane NAC, and trastuzumab if HER2-positive. Three hundred sixty-five achieved pCR (59/537 HR-positive/HER2-negative, 158/301 HER2-positive, 148/309 TNBC). CNS recurrence occurred in 72 (6.2%) patients, with no difference between pCR and non-pCR (4.7 vs. 7.0%, *p* = 0.15). Across subtypes, there was no difference for HR-positive/HER2-negative (3.4 vs. 4%, *p* = 1.0), TNBC (5.4 vs. 9.3%,* p* = 0.2), however there was a reduction in HER2-positive (4.4 vs. 14.7%, *p* = 0.003) after pCR. Isolated CNS relapse was the predominant pattern of CNS metastasis (82.4%) in pCR, particularly in HER2-positive. Median OS after CNS relapse was 12 months. Multivariate analysis identified HER2-positive, TNBC, and cN2–3 status as independent predictors of CNS recurrence.

**Conclusion:**

Although pCR was not associated with a lower overall risk of CNS recurrence, it predicted a reduced risk in HER2-positive disease. Isolated CNS relapse predominated, suggesting a sanctuary effect.

**Supplementary Information:**

The online version contains supplementary material available at 10.1007/s10549-026-07915-7.

## Introduction

Pathological complete response (pCR), defined as the absence of invasive residual disease in the breast or lymph nodes (ypT0/is ypN0) following neoadjuvant chemotherapy (NAC), is associated with significantly improved outcomes in patients with breast cancer, including a reduced risk of distant recurrence and prolonged overall survival [1]. The prognostic value of pCR is especially pronounced in triple-negative breast cancer (TNBC) and HER2-positive subtypes, and evidence suggests it also has predictive value in high-grade hormone receptor-positive/HER2-negative tumors [2].

Despite its favorable prognostic implications, up to 20% of patients who achieve pCR experience disease recurrence within 5 years [3, 4]. The intensification of NAC regimens—such as the addition of immunotherapy in TNBC and pertuzumab in HER2-positive disease—has contributed to higher pCR rates and improved survival outcomes. However, even among patients receiving optimal systemic treatment, recurrence rates still fall between 5 and 15% after pCR in more contemporaneous cohorts [5–8].

Sites of disease recurrence in this population remain insufficiently characterized in clinical trials. Due to the blood–brain barrier, chemotherapy and monoclonal antibodies are thought to have limited penetration in the central nervous system (CNS), and pCR after NAC may not correlate with a reduced risk of CNS relapse. Retrospective studies suggest that brain metastases account for a notable proportion of those recurrences, particularly in patients with HER2-positive or triple-negative disease [3, 9–11]. Nevertheless, comparative analyses of patterns of recurrence between patients who achieve pCR and those who do not remain scarce [12]. Thus, we hypothesized that patients achieving pCR following NAC would still have a high rate of CNS relapses.

A deeper understanding of the risk of CNS involvement after pCR is essential for optimizing post-treatment surveillance and identifying patients who may benefit from tailored therapeutic strategies. This study aims to characterize disease recurrence, with a particular focus on CNS metastases, in a cohort of breast cancer patients treated with NAC.

## Methods

### Study design and participants

This was a retrospective cohort from a single cancer center (A. C. Camargo Cancer Center, São Paulo, Brazil) that included all consecutive early breast cancer patients treated with neoadjuvant chemotherapy from January 2007 to December 2018. Follow-up data were last updated in May 2023.

Eligible patients included those with histologically proven invasive breast carcinoma, age ≥ 18 years, with clinical stage I to III, treated with surgery with curative intent. Patients were excluded if they had insufficient data about tumor characteristics, treatment received, or follow-up, or if they had synchronous or metachronous invasive breast cancer or another synchronous invasive carcinoma. Pathology review was not systematically performed. Therefore, cases with discordant immunohistochemistry (IHC) results from pre-treatment biopsies and surgical specimens, without internal review, or patients with multicentric heterogeneous tumors were also excluded.

Breast cancer subtype was defined by IHC for hormonal receptors (HR) and HER2, and/or in situ hybridization (ISH) when HER2 staining was + 2, obtained from pre-treatment biopsy specimens. IHC was performed on surgical specimens at the treating physician’s discretion. ASCO/CAP guidelines were used for classification and were considered HR-positive if estrogen (ER) and/or progesterone (PR) receptors were ≥ 1% without HER2 superexpression/amplification (HER2 IHC 0 or + 1, or + 2 without HER2 amplification by ISH); triple-negative (TNBC), if ER < 1% and PR < 1% without HER2 superexpression/amplification; or HER2-positive, if HER2 + 3 or HER2 + 2 with ISH amplification [13–16].

The following characteristics were retrieved from medical records: age at diagnosis, gender, menopausal status, histological subtype and grade, TNM staging and anatomic stage groupings, treatments offered (chemotherapy, anti-HER2 therapy, endocrine treatment, surgery, and radiotherapy), pathological response to neoadjuvant chemotherapy, sites of first disease recurrence and whether the recurrence was in the brain-only or not (brain plus systemic recurrence).

Treatments were offered according to the assistant physician’s choice and followed the local and standard of care guidelines for breast cancer. Patients should have received at least one cycle of the planned neoadjuvant chemotherapy. HER2-positive patients were included if they had received at least one dose of trastuzumab ± pertuzumab, either in the neoadjuvant or adjuvant setting. Dose-dense chemotherapy consisted of administering doxorubicin and cyclophosphamide (AC) every 2 weeks. Brain imaging was not mandatory for staging or follow-up and was performed if clinically indicated, although CNS scanning was routinely performed for triple-negative and HER-positive tumors. This study was approved by the institutional Internal Ethics Review Board (registration number 21308019.9.0000.5432).

### Objectives and statistical analysis

The primary objective of this study was to evaluate the impact of pCR on the risk of central nervous system (CNS) recurrences in the different breast cancer subtypes. pCR was defined as no invasive carcinoma in the breast (ypT0/is) and in axillary lymph nodes (ypN0) at the time of surgery. Parenchymal brain lesions and leptomeningeal metastasis were accounted as CNS recurrences.

The secondary objectives were to evaluate the impact of pCR on other sites of disease recurrences and patterns of CNS recurrence (brain/leptomeningeal only versus synchronous systemic and brain/leptomeningeal metastasis). Other objectives were to evaluate the CNS relapse-free survival (CNS–RFS) according to pCR and breast cancer subtype, explore factors associated with the risk of CNS recurrence, calculate median time to CNS relapse, and estimate overall survival after CNS recurrence (CNS–OS).

CNS-RFS was defined as the time from breast cancer diagnosis to the time of the appearance of CNS metastasis. The median time to CNS relapse was calculated from the breast cancer diagnosis to the time of the appearance of the first CNS metastasis, in patients who recurred in the brain. CNS–OS was determined from the date of CNS metastasis diagnosis to the time of death by any cause.

Descriptive statistics were used to report clinical-pathological and demographic variables. Fisher’s Exact test and Pearson’s Chi-square test were used to evaluate differences in CNS and other recurrence sites rates between patients with or without pCR. Kruskal–Wallis and Mann–Whitney tests were used to explore differences between continuous variables.

Survival curves were generated using the Kaplan–Meier method and were compared using the log-rank test. The median follow-up time was calculated using the inverse Kaplan–Meier method. Median survival times and 95% confidence intervals (95% CI) were also reported. Tests were considered statistically significant when *p*-values (two-tailed) < 0.05. Baseline clinicopathological and demographic variables associated with CNS relapse (Wald’s *p*-value < 0.05) in the univariate analysis were selected for inclusion in the multivariate logistic regression model to assess the risk of CNS metastasis. All statistical analyses were performed with the software SPSS 23.0 (SPSS, Chicago, IL) and R Studio (v.2024.04.2).

## Results

### Population and treatment patterns

**Fig. 1:**
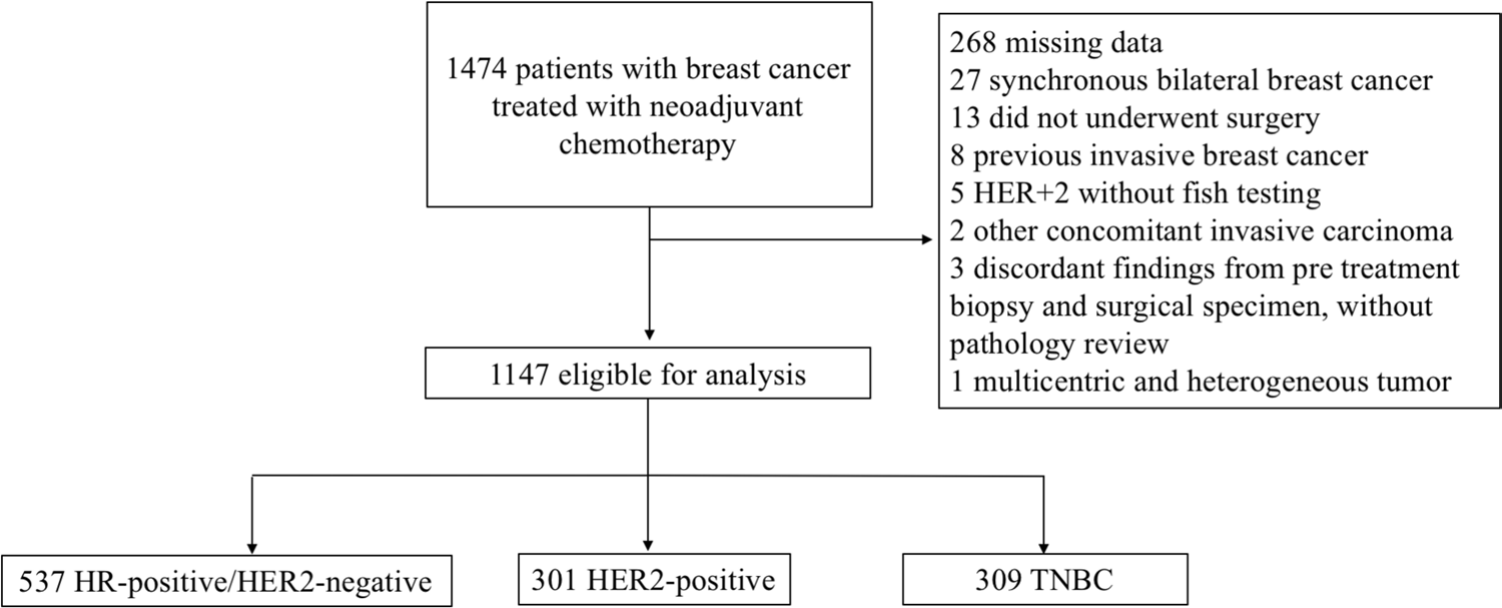
Flow diagram showing reasons for patient selection and exclusion from the study

Between January 2007 and December 2018, 1474 patients treated with neoadjuvant chemotherapy were screened, and 327 were excluded for various reasons, as described in Fig. 1, leaving 1147 eligible patients. Five hundred thirty-seven (537) patients had HR-positive/HER2-negative, 301 HER2-positive, and 309 triple-negative breast cancer. The median age was 45 years, and 61.6% of the participants were premenopausal (Table 1). Most patients had tumors with ductal histology (84.7%), grade II (48.6%) or III (42.9%), and locally advanced carcinomas (59.2% stage III).

**Table 1:** Baseline characteristics of patients in the entire cohort and each immunohistochemical subgroup

Characteristics	Total (*N* = 1.147)	HR +/HER2- (*N* = 537)	HER2 + (*N* = 301)	TNBC (*N* = 309)	*p*-value
Median age (range)	45 (23–90)	46 (24–82)	46 (23–82)	44 (24–90)	0.03
Menopausal status (%)					
Premenopausal	707 (61.6)	342 (63.7)	174 (57.8)	191 (61.8)	0.24
Postmenopausal	392 (34.1)	178 (33.1)	110 (36.6)	104 (33.7)	
Perimenopausal	45 (4)	15 (2.8)	16 (5.3)	14 (4.5)	
Male	3 (0.3)	2 (0.4)	1 (0.3)	0	
Histology (%)					
Ductal	971 (84.7)	444 (82.7)	261 (86.7)	266 (86.1)	0.001
Lobular	86 (7.5)	57 (10.6)	19 (6.3)	10 (3.2)	
Other	86 (7.5)	36 (6.7)	21 (7)	29 (9.4)	
Missing	4 (0.3)	0	0	4 (1.3)	
cT (%)					
*T*1	33 (2.9)	13 (2.4)	5 (1.7)	15 (4.9)	<.001
*T*2	425 (37.1)	171 (31.9)	111 (36.9)	143 (46.3)	
*T*3	339 (29.6)	166 (30.9)	89 (29.6)	84 (27.2)	
*T*4	350 (30.4)	187 (34.8)	96 (31.8)	67 (21.6)	
cN (%)					
*N*0	343 (29.9)	164 (30.5)	73 (24.3)	106 (34.3)	0.07
*N*1	538 (46.9)	255 (47.5)	153 (50.8)	130 (42.1)	
*N*2	189 (16.5)	88 (16.4)	55 (18.3)	46 (14.9)	
*N*3	77 (6.7)	30 (5.6)	20 (6.6)	27 (8.7)	
Clinical stage (%)					
I	15 (1.3)	2 (0.4)	2 (0.7)	11 (3.6)	<.001
II	453 (39.5)	190 (35.4)	116 (38.5)	147 (47.6)	
III	679 (59.2)	345 (64.2)	183 (60.8)	151 (48.8)	
Histologic Grade (%)					
I	55 (4.8)	46 (8.6)	6 (2)	3 (0.9)	<.001
II	557 (48.6)	325 (60.5)	155 (51.5)	77 (24.9)	
III	492 (42.9)	152 (28.3)	131 (43.5)	209 (67.7)	
Missing	43 (3.7)	14 (2.6)	9 (3)	20 (6.5)	
Hormone receptor status (%)					
ER +/PR +	589 (51.4)	437 (81.4)	152 (50.5)	0	<.001
ER +/PR–	103 (9)	70 (13)	33 (11)	0	
ER-/PR +	41 (3.6)	30 (5.6)	11 (3.7)	0	
ER-/PR–	414 (36)	0	105 (34.8)	309 (100)	
Neoadjuvant chemotherapy (%)					
Anthracycline/taxane	1090 (95)	519 (96.6)	270 (89.7)	301 (97.4)	<.001
Dose-dense AC^a^	379 (33)	149 (27.7)	67 (22.3)	163 (52.8)	<.001
Carboplatin^a^	206 (18)	17 (3.2)	28 (9.3)	161 (52.1)	<.001
Neoadjuvant anti-HER2 therapy (%)					
Trastuzumab	188 (16.4)	0	188 (62.5)	0	<.001
Trastuzumab + Pertuzumab	104 (9.1)	0	104 (34.5)	0	
No neodjuvant anti-HER2 therapy^b^	855 (74.5)	537 (100)	9 (3)^b^	309 (100)	
Adjuvant endocrine therapy (%)					
Tamoxifen	224 (19.5)	153 (28.5)	68 (22.6)	2 (0.6)^c^	<.001
AI	196 (17.1)	145 (27)	49 (16.3)	2 (0.6)^c^	
TMX/AI switch	134 (11.7)	103 (19.2)	31 (10.3)	0	
OFS + AI/Tamoxifen	163 (14.2)	118 (21.9)	45 (14.9)	0	
Refused ET	3 (0.3)	3 (0.6)	0	0	
No ET due to low HR expression	19 (1.7)	15 (2.8)	3 (1)	0	
No ET	408 (35.5)	0	105 (34.9)	304 (98.4)	
Adjuvant anti-HER2 therapy (%)					
Trastuzumab	270 (23.5)	4 (0.7)^d^	266 (88.4)	0	<.001
Trastuzumab + Pertuzumab	24 (2.1)	0	24 (8)	0	
TDM1	4 (0.4)	0	4 (1.3)	0	
No adjuvant anti-HER2 therapy^e^	849 (74)	533 (99.3)	7 (2.3)^e^	309 (100)	
Adjuvant Capecitabine (%)	60 (5.2)	14 (2.7)	0	46 (14.9)	<.001
Radiotherapy (%)	1089 (94.9)	515 (95.9)	280 (93)	294 (95.1)	0.2
Surgery (%)					
Mastectomy	827 (72.1)	416 (77.5)	214 (71.1)	197 (63.8)	<.001
Breast conserving	320 (27.9)	121 (22.5)	87 (28.9)	112 (36.2)	
Axillary dissection (%)	779 (67.9)	392 (73)	200 (66.4)	187 (60.5)	0.001

^a^The sum is greater than 100% because patients treated with dose dense AC or carboplatin are included in those that were treated with anthracyclines/taxanes

^b^Nine patients with HER2-positive breast cancer did not receive neoadjuvant trastuzumab. Among those, 6 did not have access to neoadjuvant trastuzumab and 3 received adjuvant trastuzumab after results from pathology review. All 9 patients completed 18 cycles of adjuvant trastuzumab, therefore were included in the analysis

^c^Four patients with TNBC on pre-treatment breast biopsies had residual disease with HR-positive breast cancer and received adjuvant endocrine therapy

^d^Four patients with HR-positive/HER2-negative breast cancer on pre-treatment breast biopsies had residual disease with HER2-positive breast cancer and received adjuvant trastuzumab

^e^Seven HER2-positive patients did not receive adjuvant trastuzumab, 6 due to cardiotoxicity and 1 due to rapid disease progression following surgery

*HR* hormone receptor, *TNBC* triple negative breast cancer, *ER* estrogen receptor, *PR* progesterone receptor, *AC* anthracycline + cyclophosphamide, *AI* aromatase inhibitor, *TMX* tamoxifen, *OFS* ovarian function suppression, *ET* endocrine therapy, *TDM1* trastuzumab emtansine

Some differences were observed between breast cancer subtypes. HR-positive/HER2-negative patients had a higher proportion of clinical stage III tumors (64.2%) and grade II tumors (60.5%), with 81.4% of tumors exhibiting ER and PR positivity. HER2-positive patients also had more clinical stage III (60.8%) and grade II (51.5%) tumors, with 34.8% being ER and PR negative. TNBC patients had a higher proportion of clinical stage II (47.6%) and grade III (67.7%) tumors when compared to the other subgroups.

This population was primarily treated with neoadjuvant anthracycline- and taxane-containing regimens. More than 95% of HR-positive/HER2-negative and TNBC patients, as well as 89.7% of HER2-positive patients, received both drugs. Dose-dense AC administration was more common in TNBC, with 52.8% of patients receiving this scheme versus less than a third of other breast cancer subtypes, as well as carboplatin use in 52.1% of TNBC versus < 10% for the remaining. One hundred eighty-eight (62.5%) patients with HER2-positive tumors received neoadjuvant trastuzumab, and 104 (34.5%) were treated with trastuzumab and pertuzumab, with most patients (88.4%) also treated with adjuvant trastuzumab. Reasons for not receiving anti-HER2 therapy in the neoadjuvant or adjuvant setting are described in Table 1. About 10% of patients discontinued the neoadjuvant treatment for any reason in the entire cohort (Supplementary Table 1).

Pathological complete response was achieved in 365 (31.8%) patients: 59/537 (11%) of those with HR-positive/HER2-negative tumors, 158/301 (52.3%) of those with HER2-positive tumors, and 148/309 (47.9%) of those with TNBC.

### Patterns of CNS and disease recurrence

**Fig. 2:**
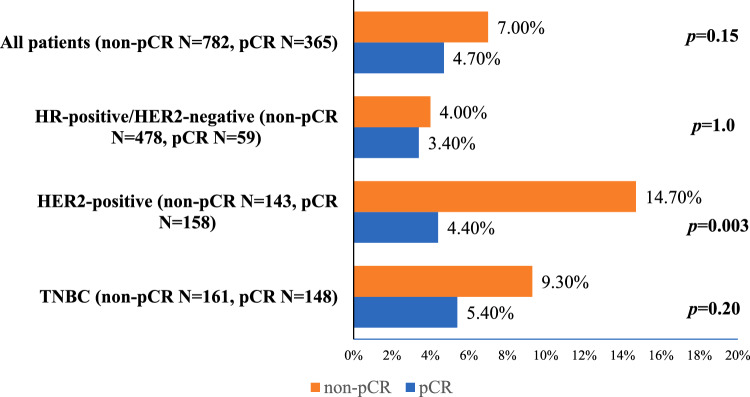
CNS recurrences by the degree of pathological response (pCR vs. non-pCR) across breast cancer subtypes. CNS recurrence occurred in a total of 72 patients. Twenty-one patients had HR-positive/HER2-negative tumors (pCR N = 2 vs. non-pCR N = 19). 28 patients had HER2-positive tumors (pCR N = 7 vs. N = 21 in non-pCR) and 23 TNBC patients (pCR N = 8 vs. N = 15 in non-pCR). pCR was defined as no invasive carcinoma in the breast and in axillary lymph nodes at the time of surgery. Frequencies were compared with Fisher’s exact test or Chi-square test

There were 72/1147 (6.2%) CNS recurrences in total, 17/365 (4.7%) occurred in patients who achieved a pCR, and 55/782 (7.0%) occurred in non-pCR patients (*p* = 0.15) (Fig. 2). Across breast cancer subtypes, patients with HER2-positive tumors who did not achieve a pCR had a threefold increase in the risk of CNS recurrence when compared with patients who achieved a pCR (14.7 vs. 4.4%, *p* = 0.003). For patients with TNBC, there was a numerically higher frequency of CNS recurrence in non-pCR patients (9.3 vs. 5.4%, *p* = 0.2). In HR-positive/HER2-negative patients, CNS recurrence was not affected by pCR. The absolute numbers of CNS recurrences were the same when considering only patients who had ypT0pN0 (excluding those with ypTispN0), in an exploratory analysis (data not shown). CNS recurrences were not impacted by neoadjuvant treatment discontinuation (Supplementary Table 2) and results were similar when considering only the cohort of patients completing all the proposed neoadjuvant treatment (Supplementary Fig. 1). Exploratory analysis showed no impact of adjuvant capecitabine on CNS recurrences in TNBC and adjuvant aromatase inhibitors had lower CNS events in HR-positive/HER2-positive patients (Supplementary Table  3 A and B).

Among the 72 CNS recurrence cases, 38 were isolated CNS recurrences and 34 were composed of CNS plus systemic recurrences. Isolated CNS recurrences were the predominant pattern of CNS metastasis after pCR (14 out of 17 CNS events in this group) when compared to non-pCR (24 out of 55 CNS events in this group) (82.4 vs. 43.6%, *p* = 0.006). This pattern of solitary brain metastasis occurring after a pCR was significant among patients with HER2-positive tumors. There was also a trend of increased risk of isolated CNS relapse among TNBC patients, but not in HR-positive/HER2-negative subtype (Table 2). The rates of disease recurrence in other sites were usually lower in patients who achieved a pCR (Supplementary Table 4).

**Table 2:** Patterns of brain recurrence according to breast cancer subtype and pCR (N = 72)

Subtype		Isolated CNS recurrence	CNS plus systemic recurrence	Total CNS recurrences	*p*- value
All patients	pCR	14 (82.4%)	3 (17.6%)	17 (100%)	0.006
	Non-pCR	24 (43.6%)	31 (56.4%)	55 (100%)	
HR-positive/HER2-negative	pCR	1 (50.0%)	1 (50.0%)	2 (100%)	1.0
	Non-pCR	10 (52.6%)	9 (47.4%)	19(100%)	
HER2-positive	pCR	7 (100%)	0 (0.0%)	7 (100%)	0.007
	Non-pCR	8 (38.1%)	13 (61.9%)	21 (100%)	
TNBC	pCR	6 (75.0%)	2 (25.0%)	8 (100%)	0.19
	Non-pCR	6 (40.0%)	9 (60.0%)	15 (100%)	

Frequencies were compared with Fisher’s exact test

### Survival and CNS recurrence

**Fig. 3:**
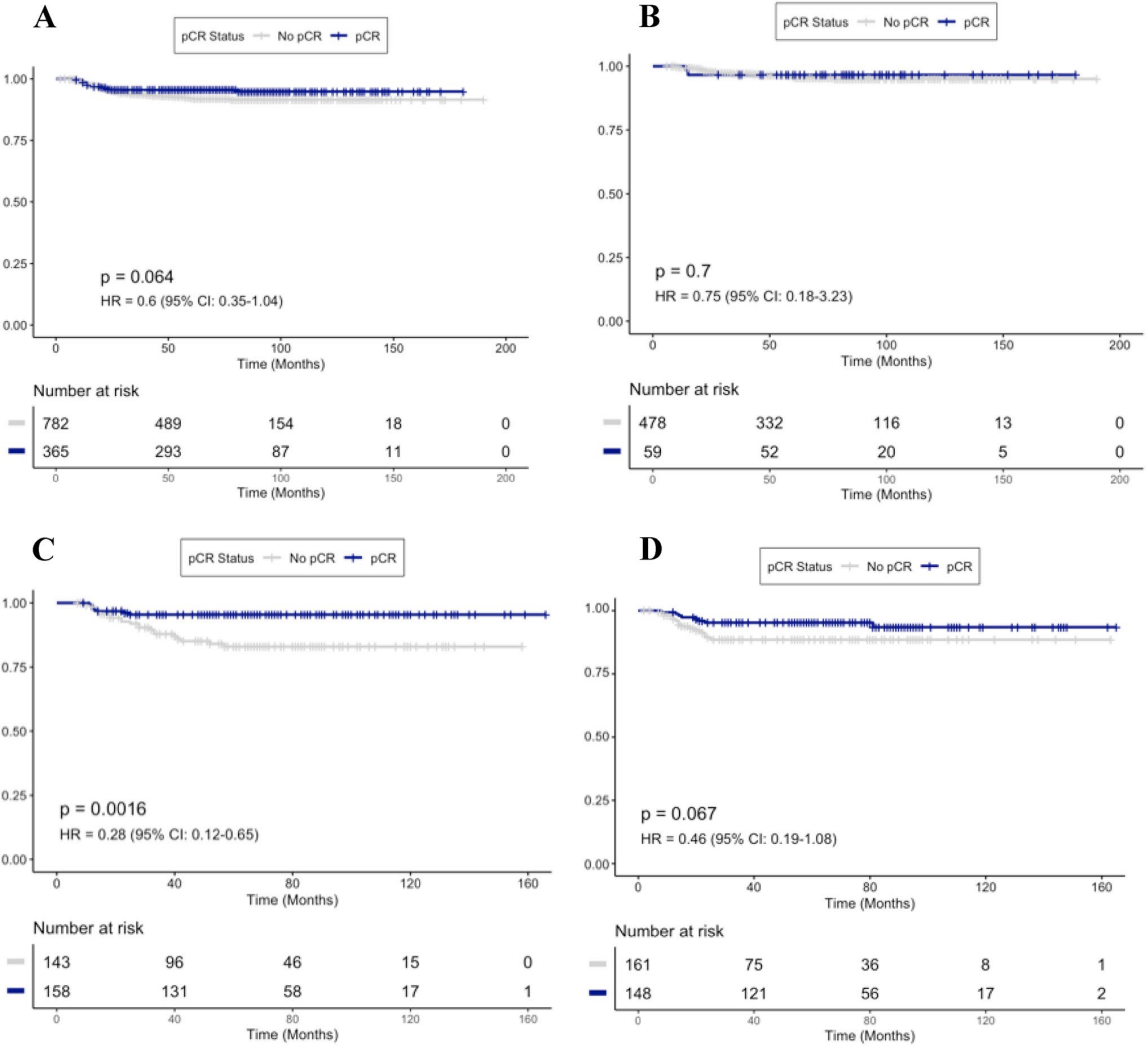
CNS–RFS according to breast cancer subtype and pCR status. **a** CNS–RFS according to pCR status in all patients, **b** CNS–RFS according to pCR in HR-positive/HER2-negative, **c** HER2-positive; and **d** TNBC

The median follow-up for the entire cohort was 81 months (95% CI 78.3–83.7). CNS-RFS was not impacted by pCR in the whole cohort (Fig. 3A). However, CNS–RFS was significantly better for HER2-positive patients who achieved a pCR (Fig. 3C). The same association was not observed in HR-positive/HER2-negative or TNBC patients (Fig. 3B and D). For patients recurring in the brain, the median time from breast cancer diagnosis to CNS relapse was 14 months (95% CI 12.0–16.0) among patients with pCR and 22 months (95% CI 19.3–24.7) among non-pCR patients, *p* = 0.14.

A univariate analysis of factors associated with CNS recurrence showed that breast cancer molecular subtype, cT, and cN stage were linked to CNS relapse risk, but not histologic grade, age, or pCR. In the multivariate analysis, HER2-positive and TNBC subtypes, and cN2 or cN3 disease were associated with significantly higher risk of CNS recurrence (Table 3).

**Table 3:** Univariate and multivariate analysis of factors associated with CNS recurrence in the entire cohort

Variable	Univariate analysis		Multivariate analysis	
	Odds ratio (95% CI)	*p*-value	Odds ratio (95% CI)	*p*-value
Histologic Grade		0.89		
I	0.32 (0.04–2.37)	0.27		
II	0.70 (0.42–1.18)	0.18		
III	1.63 (0.97–2.73)	0.07		
Subtype		0.02		
HR +/HER2-	0.48 (0.28–0.84)	0.01		
HER2 +	1.81 (1.06–3.07)	0.03	2.43 (1.34–4.40)	0.003
TNBC	1.24 (0.71–2.17)	0.44	2.10 (1.13–3.92)	0.02
cT stage		0.02		
*T*1	0.51 (0.07–3.81)	0.52		
*T*2	0.63 (0.36–1.10)	0.10	1.39 (0.18–10.8)	0.75
*T*3	0.68 (0.37–1.25)	0.22	1.30 (0.17–10.3)	0.80
*T*4	2.34 (1.39–3.92)	0.001	2.64 (0.34–20.3)	0.35
cN stage		0.002		
*N*0	0.36 (0.17–0.73)	0.01		
*N*1	0.95 (0.57–1.58)	0.83	1.89 (0.91–3.94)	0.09
*N*2	2.06 (1.15–3.70)	0.02	3.23 (1.45–7.21)	0.004
*N*3	2.41 (1.10–5.32)	0.03	4.20 (1.65–10.7)	0.003
Age		0.96		
< 35	1.16 (0.58–2.33)	0.68		
35–50	0.89 (0.54–1.49)	0.67		
51–70	1.02 (0.59–1.76)	0.95		
> 70	1.21 (0.28–5.22)	0.80		
pCR		0.15		
No	1.53 (0.85–2.74)	0.16		
Yes	0.66 (0.37–1.18)	0.16		

Factors associated with CNS recurrence with *p* < 0.05 in the univariate analysis were included in the multivariate model

The median overall survival after CNS relapse was 12 months (95% CI 6.3–17.7). CNS–OS was significantly better for patients with HER2-positive (31 months, 95% CI 12.7–49.3) and HR-positive/HER2-negative (19 months, 95% CI 0.0–39.9) tumors versus TNBC (8 months, 95% CI 4.8–11.2). Patients with isolated CNS metastasis had numerically higher OS than those with concomitant CNS and systemic metastasis (Supplementary Figs. 2 A and 1B), a trend seen across all subtypes (data not shown).

## Discussion

In this retrospective analysis, we examined a large cohort of breast cancer patients treated with neoadjuvant chemotherapy to assess whether achieving a pathological complete response (pCR) was associated with a reduced risk of central nervous system (CNS) metastases as the first site of recurrence. Our cohort predominantly consisted of patients with locally advanced and high-grade tumors, most of whom were premenopausal. HR-positive/HER2-negative and triple-negative breast cancer (TNBC) patients were primarily treated with anthracycline- and taxane-based regimens, while all HER2-positive patients received trastuzumab-based therapy. The main findings were: (1) in the overall population, pCR was not significantly associated with a lower risk of CNS metastases; however, among patients with HER2-positive tumors, pCR reduced CNS recurrence risk; (2) among patients who achieved pCR, the predominant pattern of CNS relapse was isolated brain metastasis, particularly within the HER2-positive group; (3) HER2-positive, TNBC and clinical N2/N3 disease were associated with higher risk of CNS recurrence in the overall population; and (4) CNS recurrence typically occurred early—mostly within the first 2 years of diagnosis—and was associated with poor post-recurrence survival.

These findings underscore that while pCR generally reflects effective systemic eradication of micrometastatic disease for most patients, the CNS may remain a sanctuary site, representing a unique therapeutic challenge. Indeed, pharmacokinetic studies of cerebrospinal fluid suggest that many standard breast cancer therapies—including doxorubicin, cyclophosphamide, docetaxel, carboplatin, and trastuzumab—exhibit poor CNS penetration due to limited blood–brain barrier permeability and active drug efflux mechanisms [17–21]. Interestingly, despite this theoretical limitation, we observed that in HER2-positive breast cancer, pCR was associated with a threefold reduction in the incidence of CNS metastases. Nonetheless, the continued observation of isolated brain metastases in this subgroup suggests that therapeutic agents may not fully reach or eradicate the disease in the CNS. Alternatively, treatment may select for resistant clones with a higher CNS tropism.

Our findings partially diverge from those reported by Ferraro et al. who conducted a retrospective study of 526 HER2-positive breast cancer patients treated with NAC plus trastuzumab and pertuzumab [12]. After a median follow-up of 3.2 years, they found that pCR was associated with fewer overall disease-free survival events, except for brain-only metastasis. The cumulative incidence of CNS metastasis was similar between groups—2.4% in the pCR group versus 2.0% in the non-pCR group—although CNS recurrences occurred later in the pCR group (median 19 vs. 6.5 months, respectively). The lower incidence of brain metastases in Ferraro et al.’s cohort, when compared to the present study, may be attributable to a larger proportion of patients with earlier, node-negative disease, which may have reduced the overall risk of CNS relapse, limiting the ability to detect a difference between patients with and without pCR. Even though pertuzumab use was not associated with fewer CNS recurrences in the APHINITY trial [22], it delayed CNS progression in the CLEOPATRA trial [23], and its widespread use in their cohort could also have contributed to the differences observed.

Although clinical trial data on recurrence patterns after pCR remain limited, few studies suggest a higher incidence of brain metastases as the first site of relapse among those patients. In the EORTC 10994/BIG 1–00 trial, which primarily included patients with locally advanced breast cancer treated with anthracyclines and taxanes, an increased proportion of CNS metastases was observed following pCR, even after adjustment for breast cancer subtype [24]. Similarly, analyses from the GeparQuinto and GeparSixto trials reported a greater proportion of pCR among patients who experienced CNS relapse as the first site of recurrence [25]. In a pooled analysis of six neoadjuvant trials, CNS was the first site of recurrence in 5% of patients, comparable to the 6.2% incidence in our cohort. Additionally, pCR was not associated with a reduced risk of CNS recurrence [26].

The present study highlights the need for continued vigilance regarding signs and symptoms of CNS recurrence, even after pCR, particularly among patients with HER2-positive and TNBC who present with locally advanced disease and during the first 2 years of follow-up. Current guidelines recommend CNS imaging only in the presence of neurological symptoms in patients with stage IV or recurrent breast cancer [27]. However, the poor survival outcomes following CNS relapse underscore the urgent need for alternative strategies. Early detection approaches, such as contrast-enhanced magnetic resonance imaging (MRI) screening in asymptomatic patients with stage II–III TNBC or HER2-positive tumors, are being evaluated in a phase II trial (NCT06247449) [28]; however, routine brain surveillance remains to be investigated. Detection of circulating tumor DNA after NAC has a strong correlation with disease, preceding imaging diagnosis by several months, a strategy that could play a role in this scenario [29].

This study has several limitations. It is a single-center retrospective analysis based on medical records, with some inherent missing data; moreover, pathological review was not systematically performed. Additionally, information regarding baseline CNS imaging and whether CNS recurrences were detected through routine surveillance or symptom-driven evaluation was not available, potentially introducing bias when comparing recurrence patterns in patients with and without pCR. Standard of care treatment has also evolved over time, with the incorporation of immunotherapy for triple-negative breast cancer and dual anti-HER2 blockade with pertuzumab, which may limit the generalizability of our findings to current clinical practice. Lastly, the relatively small number of CNS events reduces the statistical power for subgroup analyses; therefore, findings should be interpreted with caution. It will be essential to assess whether immunotherapy, antibody drug conjugates, and targeted therapies with CNS activity in the advanced disease can also reduce CNS recurrences in earlier stages in ongoing clinical trials with these drugs.

## Conclusion

Pathological complete response was not associated with reduced CNS recurrence overall, but was linked to a lower risk of CNS relapse in HER2-positive breast cancer. Patients achieving pCR more frequently experienced isolated CNS relapse, suggesting a possible sanctuary effect. These findings highlight the need for tailored surveillance strategies in high-risk subtypes, particularly among patients with HER2-positive and triple-negative tumors, regardless of pCR status. More detailed data on CNS recurrence from prospective trials investigating new agents with known CNS activity in early breast cancer should be reported.

## Supplementary Information

Below is the link to the electronic supplementary material.

Supplementary file1 (DOCX 4063 KB)

## Data Availability

The data that support the findings of this study are available from the authors but restrictions apply to the availability of these data, according to Brazilian law. Data are available from the authors upon reasonable request and with permission from the institution were patients were treated and the Internal Ethics Review Board.
