# Supplementary material for: Risk of CNS relapse following pathological complete response to neoadjuvant chemotherapy in early breast cancer
Source: Breast Cancer Res Treat. 2026 Mar 23;216(3):34. doi: 10.1007/s10549-026-07915-7 (PMC13006468; doi:10.1007/s10549-026-07915-7)
Supplement: Supplementary file 1 — Supplementary file1 (DOCX 4063 KB) [file 10549_2026_7915_MOESM1_ESM.docx]

**Supplementary**

**Supplementary Table 1 – Neoadjuvant treatment discontinuation for all patients and different breast cancer subtypes.**

| **Neoadjuvant Treatment discontinuation** | **All patients**  **(N=1147)** | **HR+/HER2-**  **(N=537)** | **HER2+**  **(N=301)** | **TNBC**  **(N=309)** |
| --- | --- | --- | --- | --- |
| **Yes (%)** | 119 (10.4) | 54 (10.1) | 25 (8.3) | 40 (12.9) |
| **No (%)** | 1028 (89.6) | 483 (89.9) | 276 (91.7) | 269 (87.1) |

**Supplementary Table 2 – Neoadjuvant treatment discontinuation and CNS recurrences.**

| **Neoadjuvant Treatment discontinuation** | **CNS recurrence**  **all patients**  **(N=1147)** | | **p-value** | **CNS recurrence**  **HR+/HER2-**  **(N=537)** | | **p-value** | **CNS recurrence**  **HER2+**  **(N=301)** | | **p-value** | **CNS recurrence**  **TNBC**  **(N=309)** | | **p-value** |
| --- | --- | --- | --- | --- | --- | --- | --- | --- | --- | --- | --- | --- |
|  | Yes  (N=72) | No (N=1075) |  | Yes  (N=21) | No (N=516) |  | Yes (N=28) | No (N=273) |  | Yes (N=23) | No (N=286) |  |
| **Yes** | 8 | 111 | p=0.83 | 3 | 52 | p=0.93 | 3 | 22 | p=0.63 | 3 | 37 | p=0.99 |
| **No** | 64 | 964 |  | 25 | 464 |  | 25 | 251 |  | 20 | 249 |  |

Supplementary Table 2. Frequencies were compared with Pearson’s Chi-square test.

**Supplementary Table 3A – CNS recurrence among TNBC with residual disease (N=161) receiving or not adjuvant capecitabine**

| Adjuvant Capecitabine | CNS relapse | | p-value |
| --- | --- | --- | --- |
|  | Yes | No |  |
| Yes (N=46) | 4 (8.7%) | 42 (91.3%) | p=0.86 |
| No (N=115) | 11 (9.6%) | 104 (90.4%) |  |

**Supplementary Table 3B – CNS recurrence among HR+HER2+ patients (N=196) by different adjuvant hormone therapies received**

| Adjuvant hormone therapy | CNS relapse | | p-value |
| --- | --- | --- | --- |
|  | Yes | No |  |
| Tamoxifen (N=68) | 10 (14.7%) | 58 (85.3%) | p=0.001 |
| Aromatase Inhibitor (N=48) | 0 (0%) | 49 (100%) |  |
| TMX/AI switch (N=31) | 3 (9.7%) | 28 (90.3%) |  |
| OFS+AI (N=44) | 1 (2.3%) | 43 (97.7%) |  |
| OFS+TMX (N=1) | 1 (100%) | 0 (0%) |  |

**Supplementary Table 4. Sites of disease recurrence according to pCR among different breast cancer subtypes.**

| **Site of disease recurrence** |  | **HR+/HER2-**  **(pCR N=59;**  **non-pCR N=478)** | ***p*-value** | **HER2+**  **(pCR N=158;**  **non-pCR N=143)** |  | **TNBC**  **(pCR N=148;**  **non-pCR N=161)** | ***p*-value** |
| --- | --- | --- | --- | --- | --- | --- | --- |
| **Locoregional** | pCR | 1 (1.7%) | 0.35 | 2 (1.3%) | 0.008 | 3 (2%) | <0.0001 |
|  | non-pCR | 28 (5.9%) |  | 11 (7.7%) |  | 25 (15.5%) |  |
| **Bone** | pCR | 4 (6.8%) | 0.04 | 2 (1.3%) | <0.0001 | 2 (1.4%) | <0.0001 |
|  | non-pCR | 83 (17.4%) |  | 19 (13.3%) |  | 21 (13%) |  |
| **Visceral** | pCR | 3 (5.1%) | 0.09 | 4 (2.5%) | <0.0001 | 2 (1.4%) | <0.0001 |
|  | non-pCR | 62(13%) |  | 23 (16.1%) |  | 44 (27.3%) |  |

Supplementary Table 3. Frequencies were compared with Fisher’s exact test.

**Supplementary Figure 1 – CNS recurrences by the degree of pathological response (pCR vs. non-pCR) across breast cancer subtypes in the population completing neoadjuvant treatment (N=1028).**

***p*=0.35**

***p*=0.003**

***p*=1.0**

***p*=0.13**

Figure 1. CNS recurrence occurred in a total of 64 patients. Nineteen patients had HR-positive/HER2-negative tumors (pCR N=2 vs. non-pCR N=17), 25 patients had HER2-positive tumors (pCR N=6 vs. N=19 in non-pCR) and 20 TNBC patients (pCR N=8 vs. N=12 in non-pCR). pCR was defined as no invasive carcinoma in the breast and in axillary lymph nodes at the time of surgery. Frequencies were compared with Fisher’s exact test or Chi-square test.

**Supplementary Figure 2. Overall survival in CNS relapsing patients (CNS-OS) (N=72).**

**B**

**A**


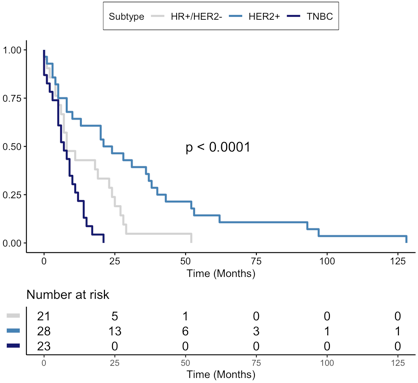

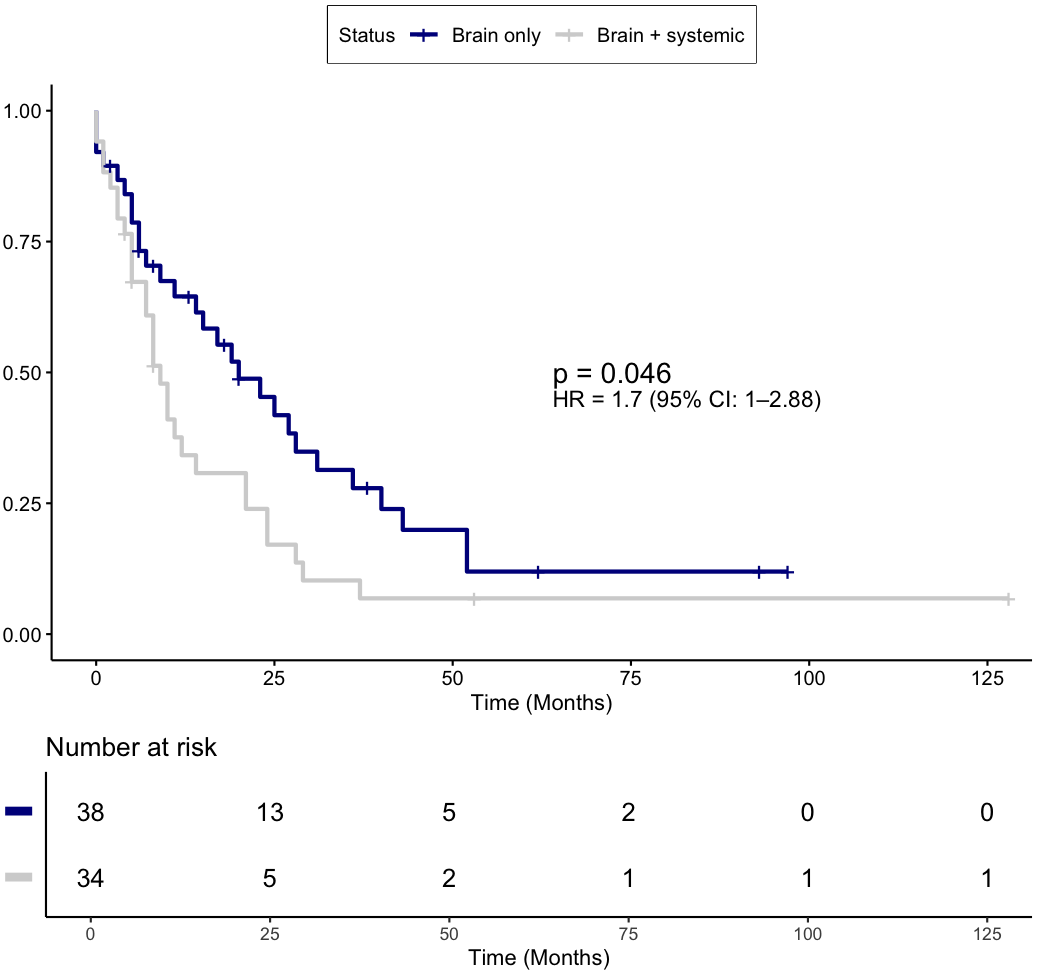


Supplementary Figure 2. A) CNS-OS according to breast cancer subtype (HR+/HER- vs. HER2+ vs. TNBC); and B) CNS-OS by pattern of CNS recurrence (brain only vs. brain plus systemic recurrence).
